# Telehealth Business Models and Their Components: Systematic Review

**DOI:** 10.2196/33128

**Published:** 2022-03-29

**Authors:** Farnia Velayati, Haleh Ayatollahi, Morteza Hemmat, Reza Dehghan

**Affiliations:** 1 Department of Health Information Management School of Health Management and Information Sciences Iran University of Medical Sciences Tehran Iran; 2 Health Management and Economics Research Center Health Management Research Institute Iran University of Medical Sciences Tehran Iran; 3 Department of Health Information Technology Saveh University of Medical Sciences Saveh Iran; 4 Department of Health Entrepreneurship Management Virtual University of Medical Sciences Tehran Iran

**Keywords:** telehealth, telemedicine, mobile health, business model, value, commerce, revenue, market, systematic review, health care

## Abstract

**Background:**

Telehealth technology is an excellent solution to resolve the problems of health care delivery. However, this technology may fail during large-scale implementation. As a result, business models can be used to facilitate commercialization of telehealth products and services.

**Objective:**

The purpose of this study was to review different types of business models or frameworks and their components used in the telehealth industry.

**Methods:**

This was a systematic review conducted in 2020. The databases used for searching related articles included Ovid, PubMed, Scopus, Web of Science, Emerald, and ProQuest. Google Scholar was also searched. These databases and Google Scholar were searched until the end of January 2020 and duplicate references were removed. Finally, articles meeting the inclusion criteria were selected and the Critical Appraisal Skills Programme (CASP) checklist was used for appraising the strengths and limitations of each study. Data were extracted using a data extraction form, and the results were synthesized narratively.

**Results:**

Initially, 4998 articles were found and after screening, 23 were selected to be included in the study. The results showed that new telehealth business models were presented in 13 studies, and the applications of the existing business models were reported in 10 studies. These studies were related to different types of services, namely, telemonitoring (4 studies), telemedicine (3 studies), mobile health (3 studies), telerehabilitation (3 studies), telehealth (2 studies), assisted living technologies (2 studies), sensor-based systems (2 studies), and mobile teledermoscopy, teleradiology, telecardiology, and teletreatment (1 study related to each area). In most of the business models, value proposition, financial variables, and revenue streams were the main components.

**Conclusions:**

Applying business models in the commercialization of telehealth services will be useful to gain a better understanding of the required components, market challenges, and possible future changes. The results showed that different business models can be used for different telehealth technologies in various health systems and cultures. However, it is necessary to evaluate the effectiveness of these models in practice. Moreover, comparing the usefulness of these models in different domains of telehealth services will help identify the strengths and weaknesses of these models for future optimization.

## Introduction

Currently, health care systems are experiencing significant increases in costs mainly due to the shortage of health care professionals, increasing life expectancy, growing elderly population, and identification of new diseases and treatment methods [1,2]. In addition, economic developments, improved quality of life, and better health conditions along with more efficient policy making have led to a demographic transition (ie, an increase in the elderly population and a reduction in the young population) [3]. To resolve the challenges associated with health care delivery to different groups of patients, information technology–based solutions such as telehealth technology have been proposed [4-6]. Telehealth is defined as the use of information and communication technology to provide a wide range of health care services [7,8]. Telehealth has also been considered a unique opportunity to bridge the gaps and inequalities in health care delivery and as a solution to reduce the pressure imposed on health care systems [9,10]. It should be noted that the term telehealth includes telemedicine, eHealth, and mobile health (mHealth), and these terms are sometimes used interchangeably [1,5,11,12].

Currently, commercialization in the telehealth industry has received significant attention and innovative technology–based start-ups are expanding. In fact, the real value of these innovations lies in their commercialization [13-17]. The results of various studies show that the use of innovative technologies in the fields of telehealth and telemedicine is very challenging, and many products in these fields either fail in the implementation phase or stop in the research and development phase [13-16]. Most of these innovations and new technologies have never been introduced at the market level, as they have mainly focused on technology-based solutions rather than real value [1,14,15]. There are also a number of nontechnical challenges such as the nature of the relationship between health care providers and patients, the responsibility of information technology professionals, and privacy and confidentiality issues that should not be underestimated [18]. To overcome these challenges, the use of business models seems inevitable for successful commercialization of innovative technologies, and it may lead to more effective and efficient provision of health care services [17].

Recently, different business models have been proposed for the telehealth industry [1,19]. However, the findings of the research conducted by Frederickson et al showed that a business model and its components should be chosen based on the purpose of the technology and the context of use [20]. The results of other studies have indicated that patients, health care providers, payers, vendors, and other stakeholders play a key role in providing telehealth and telemedicine services. If a business model provides social or economic value for all stakeholders, then the likelihood of the successful implementation of a technology will increase [13,21]. It should be noted that different business models may have different components, as reported in various studies [22,23]. As successful commercial investment in telehealth requires an appropriate business model and plan, understanding these models and their components will help technology developers and commercial investors to make more informed decisions in this field [22,23]. Therefore, the purpose of this study was to review different types of business models and their components used in the telehealth industry.

## Methods

This study was a systematic review conducted in 2020. A systematic review is a type of review in which a systematic method is used to summarize evidence on questions with a detailed and comprehensive method [24]. Before conducting this review, ethical approval was obtained from the ethics committee of the Iran University of Medical Sciences (reference number: IR.IUMS.REC.1397.1328).

### Eligibility Criteria

To select the most relevant studies, inclusion and exclusion criteria were set. According to the inclusion criteria, all research papers, reviews, conference papers, theses, and dissertations in which business models or business frameworks and their components were discussed in relation to telehealth, telemedicine, and mHealth were included in the study. No time frame was considered for searching the articles and the search was conducted until the end of January 2020. Papers published in English and full-text availability were the other inclusion criteria.

According to the exclusion criteria, books, book chapters, letters to the editor, and studies in which a business model or framework was used in fields other than telehealth, telemedicine, and mHealth were excluded. Publication languages other than English and unavailability of full texts were the other exclusion criteria.

### Information Sources

The databases used for searching articles included Ovid, PubMed, Scopus, Web of Science, Emerald, and ProQuest. Google Scholar was also searched. The searches were conducted until the end of January 2020 and duplicate references were removed. Additionally, the OpenGrey database was searched to find grey literature. The search process was carried out by reference and citation tracking, and the scientific profiles of the authors of the articles were examined to find further related articles.

### Search Strategy

To develop a search strategy, MeSH (Medical Subjects Headings) terms such as commerce, mHealth, and telemedicine and key terms such as business, business model, value chain, eHealth service, and commercial phenomena were used. The search strategies, number of records, and search dates are presented in Multimedia Appendix 1. There was no time limit for searching the articles, but the language was limited to English and only full-text papers were included in the study.

### Selection Process

The screening process was performed based on the PRISMA (Preferred Reporting Items for Systematic Reviews and Meta-Analysis) checklist [25,26]. After retrieving relevant articles, reference management was performed using EndNote software (Version X7, Clarivate) and duplicates were removed. The titles, abstracts, and full texts of the retrieved studies were screened. The initial search and screening processes were conducted by one of the authors (FV). In the next step, the other authors independently screened and appraised the remaining articles and resolved discrepancies by discussion and reached a consensus.

### Data Collection Process

Data were extracted using a data extraction form comprising the name(s) of the author(s), year of publication, country, research objective, research method, business model, the model’s components, and a summary of the results. The first author (FV) initially collected the data, and the reports were reviewed independently by other researchers too. In case of disagreement, the researchers discussed the issue and resolved it by reaching a consensus.

### Data Items

In this study, the business models or frameworks and their components used for the commercialization of telehealth services were the main data items that were reviewed and compared in different studies.

### Risk of Bias Assessment

The quality of the studies was assessed using the Critical Appraisal Skills Programme (CASP) checklist [27]. As qualitative methods were used in the selected studies, the CASP checklist for qualitative research was used. It consists of 10 questions, with “yes,” “no,” or “can’t tell” as the answer options. The calculated scores showed if the quality of each study as high (7-10), medium (4-6), or low (1-3). The assessment was conducted by 2 researchers (FV and HA) independently (see Multimedia Appendix 2).

### Synthesis Methods

In most of the selected studies, qualitative methods were used. Therefore, meta-analysis was not possible. The papers were divided into 2 groups. The first group included those studies in which a new business model or framework was presented, and the second group included papers analyzing existing business models used in the telehealth industry. To summarize data, tables were developed based on the data extraction form. The main components of the business models are also presented in figures for better understanding.

## Results

### Study Selection

**Figure 1:**
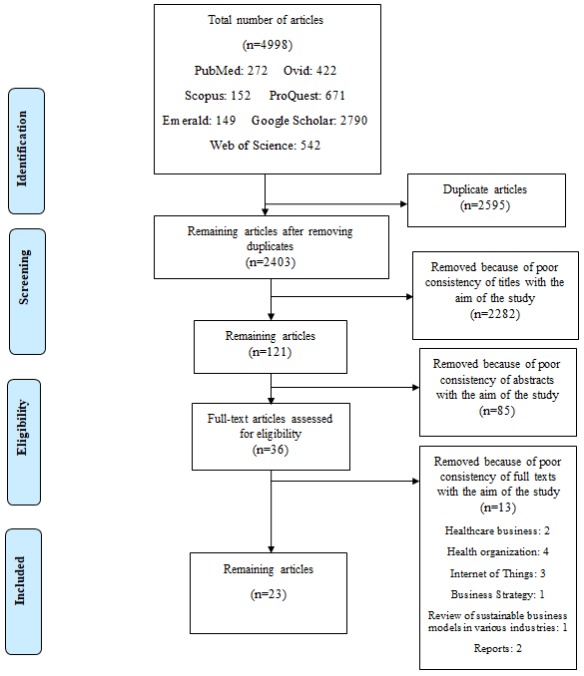
Article selection process based on PRISMA (Preferred Reporting Items for Systematic Reviews and Meta-Analyses) [25،26].

The preliminary search results in the selected databases included 4998 articles. After excluding duplicates, 2403 articles remained. Then, the titles of these articles were reviewed, and 2282 articles were excluded due to poor alignment of their aims with those of this study. In the next step, the abstracts of the 121 remaining articles were reviewed and 85 papers were excluded because their content was mostly irrelevant to the aims of this study. The full texts of the remaining articles (n=36) were reviewed, and 13 articles were excluded as they were mainly related to health care businesses (n=2), health organizations (n=4), Internet of Things (n=3), business strategy (n=1), sustainable business models in various industries (n=1), and organizational reports (n=2). Finally, 23 papers were selected to be included in this review. Figure 1 shows the article selection process.

### Study Characteristics

The papers selected for inclusion in this review were divided into 2 main groups. The first group included 13 studies that presented a new business model or framework [1,14,19,23,28-36], and the second group comprised 10 studies that evaluated existing business models or frameworks used in the telehealth industry [37-46]. These studies were published between 2005 and 2020. The first group comprised studies conducted in the United States (n=5), the Netherlands (n=1), Germany (n=2), Taiwan (n=3), Italy (n=1), and England (n=1); the second group included studies conducted in the Netherlands (n=6), China (n=1), Australia (n=2), and Sweden (n=1).

### Results of Individual Studies

#### New Business Models or Frameworks for Telehealth Industry

Table 1 presents the 13 studies analyzing a new business model or framework for use in the telehealth industry [1,14,19,23,28-36].

**Table 1:** Summary of the studies presenting a new business model or framework for use in the telehealth industry.

Author(s) (year) and country	Objective	Methods	Business model	Model components	Results
Barker et al (2005) [23] United States	To describe a business model that was developed specifically to distribute telemedicine services throughout the state of Arizona at the lowest possible cost	Qualitative study (case study)	Arizona Telemedicine Program (ATP) business model	Five components: vendor services layer, infrastructure services layer, operational services layer, professional services layer, and client layer	The ATP business model was a layered model where each layer supported the upper layer, and the membership model has allowed the ATP to develop a modern telecommunication network that delivers services to clients at a lower cost because of its distributed network and services.
Mun et al (2005) [28] United States	To provide a business model of teleradiology	Qualitative study (literature review)	Teleradiology business model	Five teleradiology business models: stand-alone teleradiology practice, the “nighthawk”/on-call coverage, solo radiologist practice, expert/second-opinion teleradiology, and a global virtual radiology service based on workload sharing and reallocation	This successful business model will depend on the ability to produce the highest-quality product at the lowest cost.
Fife and Pereira (2008) [30] United States	To provide a VISOR^a^ business model framework for mobile telehealth	Qualitative study (case study)	VISOR business model framework	Five components: value proposition, interface, service platforms, organizing model, and revenue model	The VISOR framework suggests that widespread adoption of mobile health care can only be achieved when the interface, service platform, organizational model, and revenue model are addressed simultaneously.
Kijl et al (2010) [14] Netherlands	To design a business model for a myofeedback-based teletreatment service (MyoTel) in patients with chronic neck and shoulder pain or whiplash injury	Mixed methods study (quantitative and qualitative case study)	Abstract cost benefit model (ACBM)	Two components: demand and supply	The business model engineering strategy provided important insights that led to an improved, a more viable, and a feasible business model; the related value network design and the process of early-stage business model engineering reduce risk and produce substantial savings in costs and resources related to service deployment.
Lin et al (2010) [32] Taiwan	To analyze the business model of a service innovation case by evaluating a telecardiology service	Qualitative study (case study)	Telecardiology business model	Nine components: value proposition, target customer, distribution channel, (customer) relationship, value configuration, capability, partnership, cost structure, and revenue model	The telecardiology service continued to succeed because of the mutual benefits it offered to the providers and users. A telecare service is meaningful to the general public only when the business model is sustainable.
Lin et al (2011) [33] Taiwan	To generate a framework to analyze 6 major telemedicine projects in Taiwan	Qualitative study (interviews with hospitals, security firms, and not-for-profit organizations)	Telemedicine framework	Six components: value proposition, target customers, service process, resources and capabilities, partnership and cost structure, and revenue model	Value proposition, partnership, resource, and capability affect service processes and cost structures. This in turn impacts customers’ acceptance of telemedicine.
Fachinger and Schöpke (2014) [34] Germany	To identify, describe, and develop business models of sensor-based fall detection systems	Qualitative study (literature review)	Consistent business model	Nine partial models: customer, market, financing, proceeds, production, resources, procurement, network, and strategy	A sustainable business model was built by interconnecting 9 partial business models.
Peters et al (2015) [19] Germany	To describe, analyze, and classify a business model	Qualitative study (literature review)	CompBizMod framework	Four components: value proposition, value co-creation, value communication and transfer, and value capture	This business framework was useful for coordinating the perspectives of different telemedicine institutions, evaluating competitors, and designing competitive advantages.
Fusco and Turchetti (2015) [31] Italy	To identify the best business model to optimize value creation for most project stakeholders	Qualitative study (interviews with decision makers, physiotherapists, patients, and caregivers)	Telerehabilitation business/governance models	Three components: key activities, customer/patient segments, and key resources	Telerehabilitation business models reduced costs and the number of people on the waiting list. Actually, due to changes in the health sector and innovative governance, patients can be involved in the recovery process.
Lee and Chang (2016) [29] Taiwan	To find a business model to improve the health management of patients with chronic kidney disease	Qualitative study (literature review)	Mobile health management business model	Four components: data, data analysis/service, user, and partner	Requirement analysis and design of the mobile health management business model led to the provision of a cheap and professional support and management services platform for the disease.
Oderanti and Li (2016) [35] England	To investigate the commercialization of assisted living technologies and provide a sustainable business model	Qualitative study (literature review)	Sustainable business model	Seven components: value proposition, product innovation and commercialization, infrastructure management, customer relations management, financial viability and sustainability, stakeholder credibility, and revenue streams	The comparative advantage of a sustainable business framework was the most important factor that encouraged older people to pay for eHealth despite their free health services. Further, this sustainable model reduced the pressure on the British health system.
Pereira (2017) [1] United States	To identify the value proposition of telehealth	Qualitative study (literature review)	VISOR business model	Five components: value proposition, interface, service platforms, organizing model, and revenue model	The VISOR framework illustrates that although technology issues, such as security and privacy considerations, remain key factors that will determine the rate of adoption of telehealth, nontechnological challenges are equally, if not more, important.
Arkwright et al (2019) [36] United States	To provide a business model for the success of telehealth programs	Qualitative study (literature review)	Telehealth business model	Eight models: direct-to-consumer (patient), organization-to-organization, clinician-to-clinician, oversight and processes, online patient access/portals/technology, mHealth/medical applications, hardware/software, and international telehealth program	A successful telemedicine business model must be safe, appropriate for the patient’s needs, patient-centered, user-friendly, compliant, mission driven/strategically aligned, and have demonstrable value for the patient.

^a^VISOR: value proposition, interface, service platform, organizing model, and revenue.

The study by Barker et al [23] presents a 5-layer model for telemedicine. From the bottom to the top, these layers included the vendor services layer, infrastructure services layer, operational services layer, professional services layer, and client layer. In this model, each layer supported the top layer, and the model created a new and low-cost infrastructure for telecommunication by developing a membership program and connecting to other networks. It also led to the distribution of specialized clinical services in rural communities [23]. Mun et al presented 5 business models for teleradiology including stand-alone teleradiology practice, the “nighthawk” or on-call coverage, solo radiologist practice, expert or second-opinion teleradiology, and a global virtual radiology service based on workload sharing and reallocation. These models led to more effective, higher-quality, and less-expensive diagnoses [28].

In 2 studies, business models were presented for mHealth services [29,30]. Among these, the study conducted by Fife and Pereira used the 5-component VISOR (value proposition, interface, service platform, organizing model, and revenue) model as the analytical framework to identify and address barriers to the widespread use of telehealth [30]. Another study was conducted by Lee and Chang that provided designing a 4-component business model for mHealth services for chronic kidney disease. The 4 components of this model were data, data analysis/service, partner, and user, which finally provided a cost-effective and professional platform for disease support and management services [29].

In the field of telerehabilitation, 2 different business models were presented in 2 of the included studies [14,31]. In the study by Kijl et al, a business model was considered for treating patients with chronic pain in the shoulder and neck. The design of this business model included a demand component on one side and a supply component on the other side. Medical research and development organization, occupational health care organization, and disability insurance organization were the subsets of supply and demand. In the value network of this business model, the components were interrelated, and increased productivity compensated for the additional costs of information technology [14].

Fusco and Turchetti also presented 4 models of business governance for telerehabilitation after total knee replacement. These models included 1 conservative model, 2 partnership models between primary care units and supporting companies that supplied equipment for primary care units, and 1 model based on cooperation between stakeholders. The results showed that the innovation structure was enhanced from the first to the fourth business model. The main components of these models were key activities, customer and patient segments, and key resources. These models reduced costs and the number of people on the waiting list [31].

In telecardiology, the results of the study by Lin et al showed that using a sustainable business model with the 9 components including value proposition, target customer, distribution channel, (customer) relationship, value configuration, capability, partnership, cost structure, and revenue influenced the acceptance of technology by the general public and provided mutual benefits for service providers and patients [32].

In 2 studies, a business framework for telemedicine was presented [19,33]. In the study of Lin et al, the business framework included the components of value proposition, partnership, resources and capabilities, and geography [33]. In another study, Peters et al revealed that the CompBizMod framework in telemedicine created a new perspective for reviewing and evaluating current business models in terms of structure, logic, and value. This framework included 4 main components of value proposition, value co-creation, value communication and transfer, and value capture, and the framework could be used to generate different perspectives in telemedicine business models, evaluate competitors, and determine competitive advantages [19].

The results of the study conducted by Fachinger and Schöpke showed that a sustainable business model in sensor-based fall recognition systems consists of 9 interconnected components, building blocks, or partial models including customer, market, financing, proceeds, production, resources, procurement, network, and strategy; the combined application of these components led to the creation of a sustainable business model [34]. Oderanti and Li presented a conceptual framework for a sustainable business involving assisted living technologies that included value proposition, product innovation and commercialization, infrastructure management, customer relation management, financial viability and sustainability, stakeholder credibility, and revenue streams as the 7 components. The comparative advantage of this framework was the most important factor that encouraged older people to pay for eHealth services, even though health services were free [35].

In 2 other studies, the new business models were slightly modified [30,36]. In Pereira's study, the 5-component VISOR interactive business model was the same as that presented in Fife and Pereira’s study [30], but it was presented in more detail. This model had 5 components, namely value proposition, interface, service platform, organizing model, and revenue, and the results of the study showed that the weakness of one component could be compensated by strengthening another component [1].

**Figure 2:**
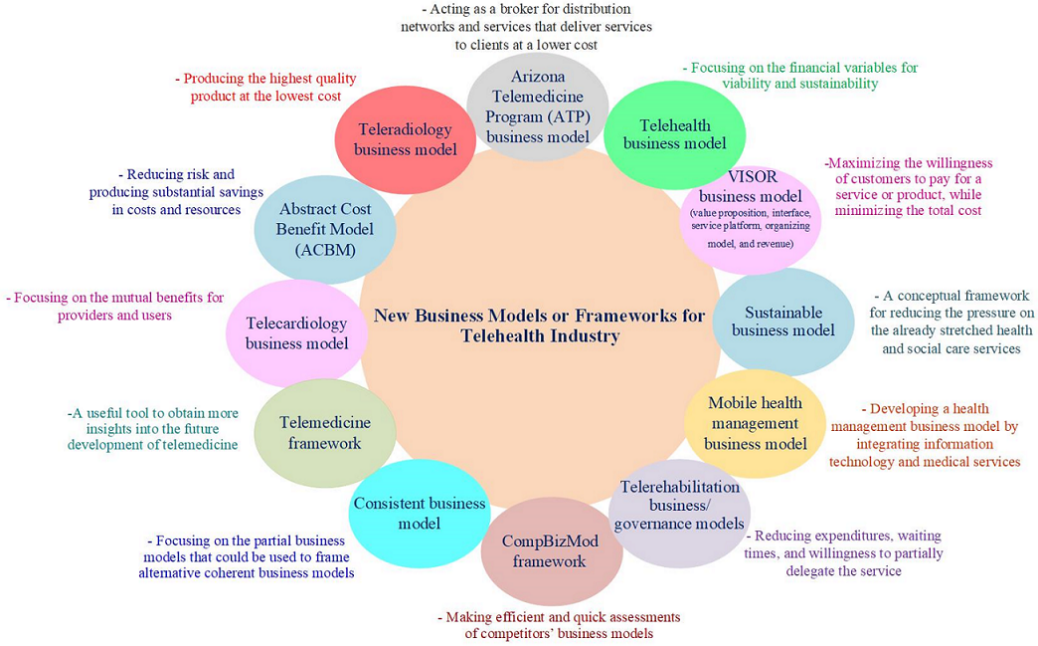
Key aspects of the new business models or frameworks for the telehealth industry.

Arkwright et al presented 8 common and successful telehealth business models in their study. These models included the direct-to-consumer (patient) business model, organization-to-organization business model, clinician-to-clinician business model, oversight and processes business model, online patient access/portals/technology, a business model based on mHealth/medical applications, a hardware/software model, and an international business model. The researchers believed that a successful telehealth business model should be safe, patient-centered, user-friendly, consistent, mission-oriented, strategy-oriented, and of proven value to the patient [36]. The key aspects of the aforementioned business models are presented in Figure 2.

#### Existing Business models or Frameworks Used in the Telehealth Industry

The findings of this study showed that 10 studies examined existing business models or frameworks used in the telehealth industry (Table 2) [37-46]. Among them, 5 studies used the 9-component Osterwalder business model, which includes customer segments, value propositions, channels, customer relationships, key resources, key activities, key partnerships, cost structures, and revenue streams [37-40,43]. Hidefjäll and Titkova showed that the development of wearable sensor technologies should be considered as part of a more extensive commercialization process consisting of conceptual, financial, and organizational developments, and the requirements of the health system should be considered [37].

The results of Marjomaa's study [38] showed that the eHealth service market for chronic diseases was a multidisciplinary market with several different segments, and the use of a participatory strategy such as the Osterwalder business model had a significant impact on the success of this market. Leeuwerden has suggested that cost-benefit studies are essential for the success of assisted living technologies in dementia care, and they can be considered along with the components of the Osterwalder model [39]. Similarly, the results of a study conducted by Kho et al showed that although the Osterwalder model can be considered as a basis for different types of telehealth businesses, the 3 components of physician participation, medical risk management, and country-specific commitments must be considered to support the sustainability of teledermoscopy services [40]. Grustam et al have stated that although attention has been paid to the components of the Osterwalder model, synergy among manufacturers, health care providers, payers, and legislators is necessary to implement telescreening technology for patients with heart diseases [43].

**Table 2:** Summary of the studies that evaluated existing business models or frameworks used in the telehealth industry.

Author(s) (year) and country	Objective	Methods	Business model	Model components	Results
Dijkstra et al (2006) [44] Netherlands	To provide a business model for telemonitoring	Qualitative study (literature review)	Freeband business blueprint method	Four components: service domain, technological domain, organizational domain, and financial domain	Using 1 flexible infrastructure for multiple telemonitoring services, infrastructure costs can be shared among multiple services. A partnership between home care organizations, central contact centers, suppliers of monitoring devices, and wireless sensor network providers is required for telemonitoring.
Leunissen (2008) [45] Netherlands	To validate the process of the business model design of Myotel (see Table 1)	Qualitative study (case study)	STOF^a^ model	Four components: service domain, technological domain, organizational domain, and financial domain	The business model is sustainable (viable, suitable for growth, and sustainable), if it has added value for all stakeholders involved.
Simonse et al (2011) [46] China	To review business models in home health services	Qualitative study (literature review)	Johnson framework	Four components: customer value proposition, profit formula, key resources, and key processes	There is an imbalance as to where money can be earned, where money can be saved, and where other value is created. Home health care providers are delivering extended, preventive, or outsourced health care from hospitals.
Marjomaa (2015) [38] Australia	To develop a generalizable business model for mHealth^b^ services in chronic disease management	Mixed methods study (quantitative and qualitative)	Alexander Osterwalder’s Business Model Canvas	Nine components: customer segments, value propositions, channels, customer relationships, revenue streams, key resources, key activities, key partnerships, and cost structure	Focusing on business model design early in the mHealth technology development phase can help researchers and designers overcome common challenges and create commercially viable mHealth services.
Hidefjäll and Titkova (2015) [37] Sweden	To design a business model for a wearable biofeedback system	Qualitative study (literature review and interviews with relevant representatives)	Alexander Osterwalder’s Business Model Canvas	Nine components: customer segments, value propositions, channels, customer relationships, revenue streams, key resources, key activities, key partnerships, and cost structure	Instead of solely focusing on the material development of the technology, development needs to be seen as part of a larger commercialization process consisting of conceptual, material, and institutional development with the business model design in focus to meet health care system requirements.
Grustam et al (2017) [42] Netherlands	To assess the B2C^c^ model for telemonitoring patients with chronic heart failure	Qualitative study (literature review)	B2C	Three components: design, structure, and governance	The B2C model in telemonitoring chronic heart failure potentially creates value for patients, who are shareholders of the service. Moreover, implementation of telemonitoring for chronic diseases via the B2C model can potentially free up financial resources, which can either be used to support a greater number of people with the same technology or can be invested in new treatments and therapies.
Grustam et al (2017) [41] Netherlands	To create the B2B^d^ and B2C care models and explore the differences in care coordination and transaction costs between these models for telemonitoring	Qualitative study (literature review)	B2B and B2C	Six components: structure, financing, public policies, technology alignment, consumers (customers), and accountability	In principle, care coordination in the B2B and B2C models for telemonitoring chronic diseases differs in terms of design elements and design themes. The transaction costs could potentially be lower in the B2C model than in the B2B model, which could be a promoting economic principle.
Grustam et al (2018) [43] Netherlands	To describe a B2C model for the implementation Of telemonitoring, by extending the current B2B model	Qualitative study (literature review)	Alexander Osterwalder’s Business Model Canvas	Nine components: customer segments, value propositions, channels, customer relationships, revenue streams, key resources, key activities, key partnerships, and cost structures	A B2B model was developed toward a B2C model offered in telemonitoring with the goal of synergizing equipment manufacturers, health care providers, payers, and legislators to enable telemonitoring for the entire population and increase the speed and scalability of the technology.
Leeuwerden (2018) [39] Netherlands	To increase the commercial viability of business model innovations with SHAAL^e^ technology in dementia care	Mixed methods study (quantitative and qualitative)	Alexander Osterwalder’s Business Model Canvas	Nine components: customer segments, value propositions, channels, customer relationships, revenue streams, key resources, key activities, key partnerships, and cost structures	Cost-benefit studies were essential to the success of ambient assisted living technology, and the insurance company played an important role in continuing to use and commercialize these technologies.
Kho et al (2020) [40] Australia	To identify, describe, compare, and contrast the building blocks for direct-to-consumer mobile teledermoscopy services	Qualitative study (literature review)	Alexander Osterwalder’s Business Model Canvas and Ash Maurya’s Lean Canvas	Nine components of Alexander Osterwalder’s business model canvas: customer segments, value propositions, channels, customer relationships, revenue streams, key resources, key activities, key partnerships, and cost structures Nine components of Ash Maurya’s Lean Canvas: customer segments, problem, revenue streams, solution, unique value proposition, channels, key metrics, cost structure, and unfair advantage	The 3 business elements that supported the viability, sustainability, and growth of web-based dermatology were developing key partnerships, clinician involvement in the design and implementation process, and managing the medicolegal risks and liabilities that are relevant for each country.

^a^STOF: service, technological, organizational, and financial.

^b^mHealth: mobile health.

^c^B2C: Business-to-Consumer.

^d^B2B: Business-to-Business.

^e^SHAAL: Smart Home and Ambient Assisted Living.

Business-to-Business (B2B) and Business-to-Consumer (B2C) models were used in 2 studies. The aim of 1 study was to explore the systemic and economic differences in care coordination via B2B and B2C models for telemonitoring patients with chronic diseases [41], and in another study, the aim was to assess the B2C model for telemonitoring patients with chronic heart failure by analyzing its value for organizations or ventures that provided telemonitoring services [42]. In these studies, the B2C model was used with its 6 components of structure, financing, public policies, technology alignment, consumers (customers), and accountability. This model created value for customers, shareholders, service providers, and the community [41,42].

Furthermore, 3 studies used other existing business models [44-46]. Dijkstra et al used the freeband business blueprint method (FBBM) including service domain, technological domain, organizational domain, and financial domain as the components. The results indicated that costs can be divided between several telemonitoring services using a flexible infrastructure [44].

**Figure 3:**
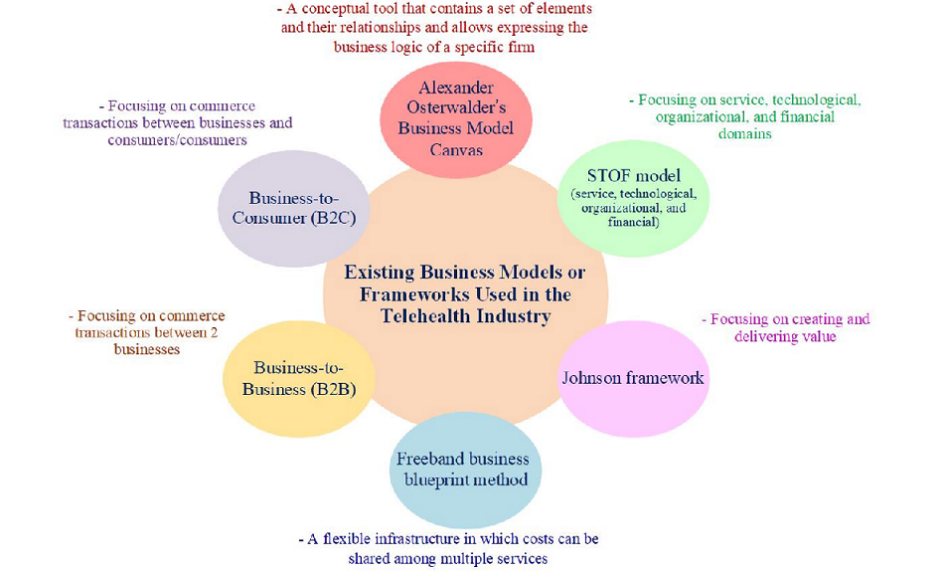
Key aspects of the existing business models or frameworks used in the telehealth industry.

In a study conducted by Leunissen, the STOF (service, technological, organizational, and financial) model was used. The results showed that the added value in the telerehabilitation business model might be changed due to the impact of cash flows [45]. In another study, Simonse et al used the Johnson framework, which included customer value proposition, profit formula, key resources, and key processes. They noted that designing a business model is not separate from the organizational context [46]. The key aspects of the existing business models or frameworks used in the telehealth industry are illustrated in Figure 3.

### Synthesis

The results showed that different types of business models and frameworks have been used in the telehealth industry and they have various components. However, value proposition, meeting the stakeholders’ and customers’ requirements, and financial issues were the most common components in these models and frameworks. These components might be described using different terms along with many other components, which were found important in relation to a specific type of technology and its context of use. Although new business models and frameworks focus on specific aspects of telehealth services, namely, service delivery, innovation, technology, and interface design along with other business components, the existing business models, such as Osterwalder’s business model canvas, have been used by some researchers to gain more comprehensive insight into the telehealth industry. It seems that using these business models and frameworks depends on the context of using the technology and many other components can be added to make them more appropriate for different purposes.

## Discussion

### Principal Findings

The aim of this study was to review different types of business models and their components used in the telehealth industry. The search process yielded 4998 articles, from which 23 studies were included in the study. These studies were divided into 2 main categories. The first category included new business models or frameworks, and the second category included the existing business models and frameworks used in telehealth industry. These models and frameworks consisted of different components in various areas of telehealth.

As mentioned earlier, business models can help implement telehealth technology with the participation of all stakeholders and in a value-based manner [4]. Business models serve as an analytical framework for identifying and overcoming barriers to the implementation and extensive use of telehealth technologies and help apply beneficial emerging technologies. These models also help identify the value proposition of telehealth services and its challenges, as well as the appropriate revenue model, organizational structure, and stakeholder engagement model [1]. However, business models must be adapted to the social, geographical, and economic contexts of the technology. Understanding each component of a business model is essential to evaluate the success of telehealth services [13,47]. Moreover, providing a business plan based on the well-known business models or frameworks, especially in the early stages of product development, will reduce potential risks and significantly save the costs related to the establishment of services and technologies [14,29,31].

A business model should be able to create and transfer value to the customers in a profitable and sustainable manner [23,30]. Therefore, some studies have emphasized the differences among the business models used for various types of telehealth technologies in each country [13,48]. For example, the results of the study conducted by Fredriksson et al showed that it is more appropriate to use different business frameworks for specific purposes. These frameworks should be in line with the context and purpose of using the technology [20]. However, the application of business models in the field of telehealth does not guarantee the success of new technologies, and before taking any action, legal issues and challenges related to licensing, compensation methods, liability, data sharing, and data protection must be resolved [28].

According to research findings, the main components in most telehealth business models were financial issues and cost structures that could be influenced by service processes, resources, and partners [33]. Cost structure plays an important role in customer acceptance, and different financial strategies need to be considered for various circumstances, revenue makings, and geographical areas [33]. Thus, a successful business model must be able to provide the highest value and increase the customers’ willingness to pay [1,36].

The results showed that it is possible to design different types of business models with various components to be used in telehealth industry. However, the components should be able to support the value of the technology in line with other components, such as the cost structure and revenue model. The components of a business model must be able to support each other, especially in unstable conditions of the health system. In addition, the components of a business model must be constantly monitored and updated [34].

A business model should ultimately lead to the acceptance of the technology by the general public. It should help in providing equitable distribution of services, effective diagnosis of diseases, and high quality of services, as well as in reducing pressure on health systems [35].

The results also revealed that some studies used existing business models or frameworks in telehealth services. Among these models, the Osterwalder business model was used more frequently than other models [37-46]. This model was helpful to meet the requirements of the health system and provided added value by increasing patient satisfaction and reducing the cost of care [37,43]. It also provided a better understanding of the business characteristics and covered various economic aspects of technology implementation [40].

A number of other studies used B2C and B2B models. The use of the B2C model allowed all stakeholders to enjoy the benefits of innovation, reduced the burden of service delivery, and improved efficiency [42]. However, when insurance companies supported the B2B model, it was more sustainable than the B2C model. Other studies used the FBBM, Johnson framework, and STOF model. The use of these models was influenced by cash flow to generate revenue and predict outcomes [44-46]. Similarly, Antoniotti et al showed that government and private payers are very influential in making telehealth payments and revenue policies should be considered in business models [49].

Although business sustainability is one of the major challenges lying ahead for the expanding telehealth industry, few studies have concentrated on this aspect [32,34,35]. The key aspects of the long-term sustainability of telehealth business include developing a skilled workforce, empowering consumers, reforming funding, improving digital ecosystems, and integrating telehealth into routine care. These requirements should be considered in implementation planning to ensure that effective integration of telehealth within complex health systems is in place and staff are willing to use telehealth technologies [50,51]. In another study, Cui et al highlighted that the sustainability of telemedicine must be improved by appropriate legislation, uniform standards, and powerful management [52].

The existing business models, especially the Osterwalder business model, are general tools and roadmaps that can provide a good understanding of business model components. However, one of its major drawbacks is the lack of sufficient emphasis on the importance of the digital economy and the functionalities of core enabling technologies. In fact, this business model cannot manage multiservice platforms and the use of other business models seems necessary to support it. Moreover, it is more product-oriented and the nature of key partner networks is less discussed in this business model [38]. In situations where the stakeholders, their roles, and the impact of their roles are different, the existing business models do not have the necessary flexibility for adaptation. Moreover, the customers (organization purchasing technology) and users (technology user) are different sometimes and considering the requirements of both groups may influence the design of the business model [53].

### Practical Recommendations

Overall, applying business models in the commercialization of telehealth services will be useful to gain a better understanding of the required components, stakeholders’ interactions, market challenges, and possible future changes. In fact, understanding the innovation, market size, competitive strategy, and investment in the telehealth industry is not sufficient and the impact of such an investment on the whole society should be investigated [38,42]. Although several business models have been proposed for use in the telehealth industry, using a combination of models and their components can help commercialize the technology more successfully. Telehealth business models can also be used in combination with traditional patient care models to double their value proposition [40,43].

### Strengths and Limitations

In this study, different types of business models and their components used in the telehealth industry were reviewed and the main components necessary for a successful telehealth business were identified. However, this study has some limitations. In most of the selected studies, qualitative approaches were used. Therefore, conducting meta-analysis was not possible. Moreover, although the main databases were searched, there might be other databases that were not searched and non-English papers that were excluded from the study. These limitations can be addressed in future studies by searching more databases and changing the exclusion criteria.

### Conclusions

The results showed that new business models used in the telehealth industry focused on legal, organizational, insurance, and customer-related issues. Added value, financial variables, financial sustainability in the market, competition, service platform, annual membership and subscription, national incentives, cost structure, and revenue streams were the other important components of these models. The studies that used existing business models mostly focused on aspects such as design, structure, governance, organizational issues, country-specific obligations, public policy, financing, profit formula, physician participation, risk management, and key processes.

In general, the diversity of business models and their components in the telehealth industry indicates that different models can be used for different telehealth technologies in various health systems and cultures. However, it is necessary to evaluate the effectiveness of these models in practice. Moreover, comparing the usefulness of these models in different domains of telehealth services will help identify the strengths and weaknesses of these models for future optimization.

## Appendix

Multimedia Appendix 1 Search strategies.

Multimedia Appendix 2 CASP (Critical Appraisal Skills Programme) checklist.

## Abbreviations

B2B: Business-to-Business
B2C: Business-to-Consumer
CASP: Critical Appraisal Skills Programme
mhealth: mobile health
FBBM: freeband business blueprint method
STOF: service, technology, organization, finance
VISOR: value proposition, interface, service platform, organizing model, and revenue
