# Supplementary material for: Telehealth Business Models and Their Components: Systematic Review
Source: J Med Internet Res. 2022 Mar 29;24(3):e33128. doi: 10.2196/33128 (PMC9006135; doi:10.2196/33128)
Supplement: Multimedia Appendix 1 [file jmir_v24i3e33128_app1.docx]

**Multimedia Appendix 1: Search strategy**

| **Results** | **Search strategy** | **Database name/ Date of search** |
| --- | --- | --- |
| **272** | Search ((business model* OR "business model framework" OR "commercial phenomena" OR commerce OR "value proposition" OR "value chain" OR "value added service" OR "service value network" OR "business strategy" OR "electronic commerce" OR "business opportunities" OR "business model innovation" OR "service business model" OR "sustainable business" OR "cost benefit analysis" OR competition)) AND (telehealth OR telemedicine OR mHealth OR "mobile health" OR eHealth OR "electronic health" OR "e-health service" OR "electronic health record" OR "medical informatics" OR teleconsultation) Sort by: Best Match | **PubMed**  31 January2020 |
| **422** | ("business model*" OR "business model framework" OR "commercial phenomena" OR commerce OR "value proposition" OR "value chain" OR "value added service" OR "service value network" OR "business strategy" OR "electronic commerce" OR "business opportunities" OR "business model innovation" OR "service business model" OR "sustainable business" OR "cost benefit analysis" OR "competition"). ab,at,kw. AND (telehealth OR telemedicine OR mhealth OR "mobile health" OR ehealth OR "electronic health" OR "e-health service" OR "electronic health record" OR "medical informatics" OR "teleconsultation").ab,at,kw. | **Ovid**  31 January 2020 |
| **542** | TOPIC: ("business model*" OR "business model framework" OR "commercial phenomena" OR commerce OR "value proposition" OR "value chain" OR "value added service" OR "service value network" OR "business strategy" OR "electronic commerce" OR "business opportunities" OR "business model innovation" OR "service business model" OR "sustainable business" OR "cost benefit analysis" OR "competition") AND TOPIC: (telehealth OR telemedicine OR mhealth OR "mobile health" OR ehealth OR "electronic health" OR "e-health service" OR "electronic health record" OR "medical informatics" OR "teleconsultation") | **Web of Science**  31 January 2020 |
| **152** | ( TITLE-ABS-KEY ( "business model*" OR "business model framework" OR "commercial phenomena" OR commerce OR "value proposition" OR "value chain" OR "value added service" OR "service value network" OR "business strategy" OR "electronic commerce" ) AND TITLE-ABS-KEY ( "business opportunities" OR "business model innovation" OR "service business model" OR "sustainable business" OR "cost benefit analysis" OR "competition" ) AND TITLE-ABS-KEY ( telehealth OR telemedicine OR mhealth OR "mobile health" OR ehealth OR "electronic health" OR "e-health service" OR "electronic health record" OR "medical informatics" OR "teleconsultation" ) ) | **Scopus**  31 January 2020 |
| **149** | (("Business model*" OR "Business model framework" OR "commercial phenomena" OR "Commerce" OR "Value proposition" OR "Value chain" OR "Value added service" OR "Service value network" OR "Business strategy" OR "Electronic commerce" OR "Business opportunities" OR "Business model innovation" OR "Service business model" OR "Sustainable business" OR "Cost benefit analysis" OR "Competition") AND ("telehealth" OR "Telemedicine" OR "mHealth" OR "mobile health" OR "eHealth" OR "Electronic health" OR "E-health services" OR "Electronic Health Records" OR "medical informatics" OR "teleconsultation")) | **Emerald**  31 January 2020 |

| **Results** | **Search strategy** | **Database name /Date of search** |
| --- | --- | --- |
| **671** | ("business model*" OR "business model framework" OR "commercial phenomena" OR commerce OR "value proposition" OR "value chain" OR "value added service" OR "service value network" OR "business strategy" OR "electronic commerce" OR "business opportunities" OR "business model innovation" OR "service business model" OR "sustainable business" OR "cost benefit analysis" OR "competition") AND (telehealth OR telemedicine OR mhealth OR "mobile health" OR ehealth OR "electronic health" OR "e-health service" OR "electronic health record" OR "medical informatics" OR "teleconsultation") | **ProQuest**  31 January 2020 |
| **2790** | (("business model*") AND ("telehealth")) | **Google scholar**  31 January 2020 |
