# Supplementary material for: Telehealth Business Models and Their Components: Systematic Review
Source: J Med Internet Res. 2022 Mar 29;24(3):e33128. doi: 10.2196/33128 (PMC9006135; doi:10.2196/33128)
Supplement: Multimedia Appendix 2 [file jmir_v24i3e33128_app2.docx]

Multimedia Appendix 2: Critical Appraisal Skills Programme (CASP) checklist

| Classification  of Quality | Score | Item 10 | Item 9 | Item 8 | Item 7 | Item 6 | | Item 5 | Item 4 | Item 3 | Item 2 | Item 1 | Author (year of publication) |
| --- | --- | --- | --- | --- | --- | --- | --- | --- | --- | --- | --- | --- | --- |
| High | 9 | Y | Y | Y | Y | | Y | Y | Y | Y | Can’t Tell | Y | Barker et al./ 2005  [23] |
| Medium | 5 | Y | Y | Y | N | N | | Can’t Tell | Y | N | N | Y | Mun et al. /2005  [28] |
| High | 9 | Y | Y | Y | Y | Y | | Y | Y | Y | N | Y | Dijkstra et al. / 2006  [44] |
| High | 9 | Y | Y | Y | Y | Y | | Y | Y | Y | N | Y | Fife and Pereira / 2008  [30] |
| High | 9 | Y | Y | Y | Y | Y | | Y | Y | Y | N | Y | Leunissen/ 2008  [45] |
| High | 10 | Y | Y | Y | Y | Y | | Y | Y | Y | Y | Y | Kijl et al. / 2010  [14] |
| High | 10 | Y | Y | Y | Y | Y | | Y | Y | Y | Y | Y | Lin et al. / 2010  [32] |
| High | 10 | Y | Y | Y | Y | Y | | Y | Y | Y | Y | Y | Lin et al. / 2011  [33] |
| High | 9 | Y | Y | Y | Y | Y | | Y | Y | Y | N | Y | Simonse et al. / 2011  [46] |
| High | 9 | Y | Y | Y | Y | Y | | Y | Y | Y | Can’t Tell | Y | Fachinger and Schöpke / 2014  [34] |
| High | 10 | Y | Y | Y | Y | Y | | Y | Y | Y | Y | Y | Peters et al. / 2015  [19] |
| High | 10 | Y | Y | Y | Y | Y | | Y | Y | Y | Y | Y | Fusco and Turchetti/ 2015  [31] |
| High | 10 | Y | Y | Y | Y | Y | | Y | Y | Y | Y | Y | Marjomaa/ 2015  [38] |
| High | 10 | Y | Y | Y | Y | Y | | Y | Y | Y | Y | Y | Hidefjäll and Titkova / 2015  [37] |
| Medium | 6 | Y | Y | Can’t Tell | Y | N | | N | Y | Y | N | Y | Lee and Chang / 2016  [29] |
| High | 10 | Y | Y | Y | Y | Y | | Y | Y | Y | Y | Y | Oderanti and Li / 2016  [35] |
| High | 10 | Y | Y | Y | Y | Y | | Y | Y | Y | Y | Y | Pereira / 2017  [1] |

| Classification  of Quality | Score | Item 10 | Item 9 | Item 8 | Item 7 | Item 6 | Item 5 | Item 4 | Item 3 | Item 2 | Item 1 | Author (year of publication) |
| --- | --- | --- | --- | --- | --- | --- | --- | --- | --- | --- | --- | --- |
| High | 10 | Y | Y | Y | Y | Y | Y | Y | Y | Y | Y | Grustam et al. / 2017  [42] |
| High | 10 | Y | Y | Y | Y | Y | Y | Y | Y | Y | Y | Grustam et al. / 2017  [41] |
| High | 10 | Y | Y | Y | Y | Y | Y | Y | Y | Y | Y | Grustam et al. / 2018  [43] |
| High | 9 | Y | Y | Y | Y | Y | Y | Y | Y | Can’t Tell | Y | Leeuwerden / 2018  [39] |
| High | 10 | Y | Y | Y | Y | Y | Y | Y | Y | Y | Y | Arkwright et al. / 2019  [36] |
| High | 9 | Y | Y | Y | Y | Y | Y | Y | Y | N | Y | Kho et al. / 2020  [40] |

Abbreviations: Y = Yes; N = No; Item 1 = Clear statement of aim; Item 2 = Suitable qualitative methodology; Item 3 = Appropriate research design; Item 4 = Proper recruiting strategy; Item 5 = Adequacy in data collection; Item 6 = Adequate relationship between researcher and participants; Item 7 = Ethical considerations; Item 8 = Rigor in data analysis; Item 9 = Clear statement of findings; Item 10 = Overall value of research (usefulness of results locally).
